# The Patent on Antitoxin

**Published:** 1898-09

**Authors:** 


### The Patent on Antitoxin.

The announcement that Professor Behring has been granted a patent as the inventor of diphtheria antitoxin will be received by the medical profession with feelings of keen disappointment. The profession of this country has always sternly discountenanced any attempt on the part of its members to make scientific achievements opportunities of personal profit. Such discoveries as the medical profession have made have been fully and freely donated to the service of suffering humanity. Professor Behring's claim to be the exclusive inventor of antitoxin not only indicates a spirit of commercialism which does its possessor no credit, but displays a disposition to assume credit for the labors of others and to make of these an occasion of personal gain which can only indicate a high degree of moral perversity.

Professor Behring claims as his invention: 1. A process "of producing diphtheria antitoxin, which consists in inoculating horses or other animals capable of being infected with diphtheria with repeated doses of diphtheria poison or living diphtheria bacilli of gradually increasing quantity and strength so as to immunize them and form in the blood a counter-poison for destroying the poison secreted by said bacilli, drawing off the blood from said animals, separating the serum from the blood corpuscles, and concentrating the former for use substantially as set forth.

"2. As a new substance, diphtheria antitoxin, consisting of the concentrated serum of the blood of animals treated with diphtheria poison and having the characteristic of immunizing test animals against infection with diphtheria, and curing them when artificially infected with diphtheria, said serum containing a counter-poison having the property of destroying the poison secreted by the diphtheria bacilli substantially as set forth."

It is almost superfluous to point out to any well-informed reader that Behring's claim to have done this is as preposterous as it is unjust. The principles upon which immunization to diphtheria was finally achieved were of gradual growth, the outcome of researches by thousands of untiring workers. The foundation of the work was undoubtedly laid by Pasteur in his method of immunizing against chicken cholera and anthrax. So long ago as 1887 Sewall immunized pigeons against the poison of rattlesnakes. He says, with genuine modesty, his work was undertaken with the hope that it might form a worthy contribution to the theory of prophylaxis, and it was a most worthy contribution. In 1887 Roux and Chamberland immunized animals against malignant edema with sterilized anthrax cultures. In 1890, the same year in which Behring and Kitasato published their results in immunizing animals against diphtheria and tetanus, Fraenkel published his results in diphtheria after treating animals by weakened germs and filtered cultures. In the clinical uses of the serum Aronson's name must not be forgotten. His serum was first used in the Children's Hospital at Berlin in 1894. The serum of Roux had been used in one of the hospitals of Paris a month earlier than Aronson's in Germany. Emerich and Aronson both dispute the priority of Behring, and the French Academy of Sciences awarded their prize for antitoxin jointly to Behring and Roux, a fact which very clearly denotes the difficulty of estimating priority of merit in a scientific struggle in which the numerous competitors were so equally distinguished.

The principle which lies at the foundation of the invention of diphtheria antitoxin, and that which underlies all serum therapeutics, is that the blood of immune animals can be used in the treatment of others. Behring did not discover this principle, and in its application he was undoubtedly anticipated by the Japanese workers. If to any single man must be ascribed the distinction of being the inventor and discoveror of the beneficent principle of immunization, the honor belongs to the immortal Pasteur.

The manufacture of antitoxin has been carried out for many years in England, France, Switzerland, Italy, Russia, and Japan, and in these countries no one has had the temerity to attempt to control exclusively its manufacture. In this country it is made by five Boards of Health and by several manufacturing firms. In this country alone has an attempt been made to monopolize its production, it being admitted that elsewhere the claims of any patentee are inadmissible.

If Professor Behring admits any merit in the work of his predecessors and contemporaries, his claim to be the exclusive inventor of diphtheria antitoxin is in contravention of all the ethics of a scientist's career. His claim is an offense against common morality. Had Simpson patented chloroform anesthesia, or had Lister patented antiseptic surgery, the world would have had two selfish emprics, and lost two medical heroes. If Behring, by the righteous judgment of mankind, can be adjudged sole and undisputed inventor of antitoxin, he has a place in the Temple of Fame for achieving the most beneficent discovery of modern times. It remains to be seen whether the temptation to be rich will overcome his ambition to be great, and whether for a tinsel crown he will barter a diadem of everlasting renown.—Advance Sheets of Editorial in *Medical Age* for August, 1898.

